# CAR-M therapy in the era of tumor immunotherapy: current research progress and engineering strategies

**DOI:** 10.3389/fimmu.2025.1723270

**Published:** 2026-01-14

**Authors:** Yi-Min Yang, Yu-Fan Ding, Yi-Yang Hu, Fan Fan, Jun-Long Zhao

**Affiliations:** 1 State Key Laboratory of Holistic Integrative Management of Gastrointestinal Cancers, Department of Medical Genetics and Developmental Biology, Fourth Military Medical University, Xi’an, China; 2 Basic Medical College, Fourth Military Medical University, Xi’an, China

**Keywords:** cancer immunotherapy, chimeric antigen receptor, logic gate, macrophage, synthetic biology

## Abstract

Chimeric antigen receptor (CAR) cellular immunotherapy has emerged as a revolutionary modality in cancer treatment. CAR-T cell therapy has demonstrated remarkable efficacy against hematological malignancies; however, its application in solid tumors is significantly constrained by inadequate tumor infiltration, a profoundly immunosuppressive tumor microenvironment (TME), and pervasive antigen heterogeneity. Conversely, macrophages — innate immune cells inherently poised within tissues — exhibit superior tumor-tropic migration, potent phagocytic capability, and a unique capacity to remodel the TME, establishing CAR-engineered macrophages (CAR-M) as a highly promising next-generation therapeutic platform. Despite this considerable promise, the clinical translation of CAR-M faces several critical bottlenecks, including heterogeneity in cell sources, challenges in manufacturing standardization, risks of on-target/off-tumor toxicity, and the dynamic, immunosuppressive nature of the TME. This review offers a systematic and in-depth analysis of the current research landscape and engineering advances in CAR-M therapy. It comprehensively details the molecular evolution of CAR-M designs, spanning from early constructs to sophisticated logic-gated circuits and innovative *in vivo* generation strategies utilizing lipid nanoparticles (LNPs). We critically evaluate the applicability and limitations of various cellular sources, such as peripheral blood mononuclear cells (PBMCs), induced pluripotent stem cells (iPSCs), and the THP-1 cell line. Furthermore, the review elucidates the multimodal antitumor mechanisms of CAR-M, including the direct “phagocytosis-presentation-activation” cascade, synergistic potential with immune checkpoint blockade, and deep reprogramming of the immunosuppressive TME. By synthesizing the latest preclinical and emerging clinical evidence, this article underscores the distinctive advantages and delineates a translational roadmap for CAR-M development. It is intended to serve as an authoritative reference for the field, providing strategic insights into intelligent receptor design, precision biomanufacturing, and rational combination therapies aimed at overcoming the enduring barriers in solid tumor immunotherapy.

## Graphical Abstract

**Figure:**
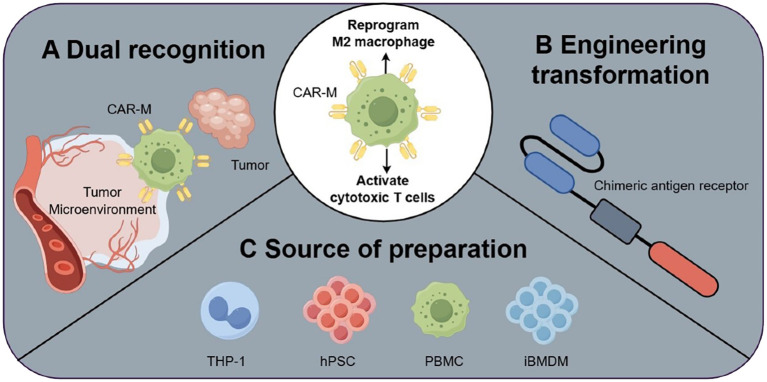
In this review, we summarize the antitumor advantages of CAR-M cells, the molecular design strategies, and the cell sources and adaptations used in the preparation process. By constructing the closed-loop logic framework of “targeting demand-molecular design-cell adaptation”, we propose its prospects for the construction of intelligent dual-environment recognition CAR-M engineering platforms on the basis of logic gating signals in the future.

## 1 Introduction

Cellular immunotherapy is a therapeutic strategy to reconstitute or enhance the antitumor immune response of patients by modifying and infusing functional immune cells *in vitro* (1). In recent years, on the basis of its personalized characteristics, controllable toxicity spectrum and relatively durable therapeutic effect, this therapy has become an important pillar of precision cancer medicine. With the breakthrough development of gene editing technologies (such as CRISPR/Cas9) (2, 3) and synthetic biology technologies (4), chimeric antigen receptor (CAR)-engineered immune cells have shown great potential in the treatment of malignant tumors. For example, the clinical efficacy of CAR-T cells in B-cell leukemia and lymphoma has been verified. At present, six CAR-T-cell therapies have been approved by the US Food and Drug Administration (FDA) (5). However, CAR-T cells face multiple biological challenges in the treatment of solid tumors, including T-cell exhaustion caused by persistent antigen exposure (characterized by abnormal expression of inhibitory receptors such as PD-1 and TIM-3 and ID3/SOX2 transcription factors) (6), as well as the synergistic inhibitory effect of immunosuppressive cell populations in the TME (such as regulatory T cells and tumor-associated macrophages) (7, 8). Together, these mechanisms lead to limited infiltration, functional inactivation and a low clinical response rate to CAR-T cells in solid tumors.

In view of this, macrophages, which naturally reside in the TME and account for up to 50% of the total population (9), have returned to the focus of research because of their unique biological characteristics. Macrophages are the core effector cells of innate immunity and can clear pathogens or malignant cells through nonspecific phagocytosis mediated by pattern recognition receptors (PRRs); they can also activate adaptive immune responses through antigen presentation, proinflammatory cytokine secretion (such as IL-12 and TNF-α) and costimulatory signal transduction (such as CD80/CD86), forming a multidimensional antitumor immune regulatory network (10–12). Thus, chimeric antigen receptor-modified macrophages (CAR-Ms) have emerged. It integrates the targeting specificity of CARs with the inherent functions of macrophages (phagocytosis, antigen presentation ability and proinflammatory cytokine secretion characteristics) to achieve efficient recognition, precise clearance of tumor cells and remodeling of the immune microenvironment (13). However, current research on CAR-Ms still faces key limitations, including the immunogenicity of allogeneic cell sources (14–16), the stability of *in vitro* polarization efficiency, the heterogeneity of TAMs (17, 18), the acclimatization of the immunosuppressive tumor microenvironment (19), and functional maintenance after cryopreservation and resuscitation (20).

This study systematically analyzes the core advantages of CAR-M therapy, focusing on its molecular design strategies (such as costimulatory domain optimization and suicide switch embedding), cell source selection (autologous vs. induced pluripotent stem cell differentiation) and engineering platforms (such as CRISPR screening-driven function enhancement) to construct a closed-loop logical framework of “targeted demand-molecular design-cell adaptation”. Moreover, this paper discusses the obstacles of TAM heterogeneity and the tumor immunosuppressive microenvironment to the practical application of CAR-M, proposes artificial intelligence-assisted intelligent gene circuit design (such as a logic-gated signaling system) and multiomics-driven precise regulation strategies, aiming to provide an innovative theoretical paradigm and a technical path for the clinical translation of CAR-M therapy.

## 2 The core advantages of CAR-M cells in tumor immunotherapy

Compared with traditional CAR-T-cell therapy, CAR-M-cell therapy has potential multidimensional biological advantages in the treatment of solid tumors by integrating the innate immune effector function of macrophages and the targeting specificity of the CAR.

### 2.1 Antigen presentation and multimodal immune activation

CAR-Ms can not only engulf tumor cells directly through Fcγ receptor (FcγR)-dependent and FcγR-independent pathways but also present unique antigen-presenting functions such as tumor-associated antigens (TAAs) to CD8+ T cells and CD4+ T cells through MHC-I/II molecules, thereby activating adaptive immune responses (21, 22). The study by Klichinsky et al. (16) is important: using the chimeric adenovirus vector Ad5f35, they engineered macrophages expressing the NY-ESO1 antigen and HLA-A2*01 and confirmed that Ad5F35-transduced CAR-M significantly induced CD8+ T-cell proliferation in coculture experiments with autologous specific T cells. Moreover, the activated T cells presented a 3-fold increase in IFN-γ secretion compared with that in the control group. These findings suggest that CAR-Ms can synergistically affect innate and adaptive immunity through the “phagocytosis, presentation, and activation” cascade to achieve deep immune surveillance of tumors.

Pierini et al. (23) An important breakthrough in the preclinical study of HER2-positive solid tumors was made: in the CT26-HER2 model with limited efficacy of PD-1 inhibitor monotherapy (only 14.3%), the complete response rate after combined CAR-M therapy significantly increased to 77.8%. The number of dendritic cells was increased approximately 6-fold in the combined treatment group, and the infiltration of CD4^+^ T cells, CD8^+^ T cells and NK cells was also significantly increased, as shown by multiple MIBI imaging. T-cell receptor beta chain (TCRB) sequencing further revealed an approximately 4-fold increase in the Morisita overlap index and a 42% decrease in the Simpson clonality index, indicating the expansion of tumor-reactive T cells *in vivo* and an increase in the diversity of the T-cell receptor repertoire. This study revealed that CAR-Ms can activate adaptive immune cells through multimodal immune activation and thus have synergistic antitumor effects with immune checkpoint inhibitors, which provides a new idea for the combined treatment of immune “cold” tumors.

### 2.2 Remodeling the immunosuppressive tumor microenvironment

TAMs usually present an immunosuppressive phenotype in the TME, which mainly drives tumor immune escape by secreting immunosuppressive factors such as IL-10 and TGF-β and promoting angiogenesis (24–26). Studies have shown that CAR-M cells can induce the reprogramming of immunosuppressive TAM subsets to a proinflammatory phenotype by secreting proinflammatory factors (such as IL-12 and TNF-α) (27, 28) and chemokines (such as CCL5 and CXCL9) (16, 29), thereby improving the immunosuppressive properties of the TME. The key evidence came from a study in HER2-positive solid tumors (16): HER2-specific CAR-M cells expressing the CD3ζ signal domain significantly reduced the proportion of immunosuppressive TAM subsets in the TME (from 71% to 14%) while increasing the expression of the proinflammatory phenotypic markers iNOS and CD86 in a humanized mouse model. Cytokine microarray analysis revealed that the secretion of the proinflammatory factor GM-CSF was significantly increased, while that of the immunosuppressive factor IL-10 was significantly decreased, and the overall transcriptional state was skewed toward the proinflammatory phenotype (30). This study confirmed the remodeling effect of CAR-M cells on the function of TAMs at the phenotypic and molecular levels. In addition, CAR-M cells can also indirectly weaken the immunosuppressive effect of regulatory T cells (Tregs) by eliminating the chemokine CCL22 from regulatory T (Treg) cells and jointly form a “two-way” microenvironmental regulatory network.

### 2.3 Potential for a universal therapeutic platform

The clinical application of CAR-T-cell therapy is limited by the need for human leukocyte antigen (HLA) matching and the risk of graft-versus-host disease (GvHD) (31, 32), whereas the low immunogenicity of macrophages provides a theoretical basis for the development of universal CAR-M (33)-cell therapy. Takata et al. (34) demonstrated for the first time that macrophages (iMACs) differentiated from induced pluripotent stem cells (iPSCs) could home to tumor tissues and differentiate into functional macrophage subsets *in vivo*. Zhang’s team (35) successfully constructed an ipSC-derived CAR-M (CAR-iMac) in 2020 and reported that it significantly inhibited the growth of ovarian cancer xenografts in an NSG mouse model. This technology solves the problems of low *in vitro* expansion efficiency and large batch heterogeneity of primary macrophages via the standardized iPSC differentiation process and lays the foundation for the large-scale production of “off-the-shelf” CAR-M. However, allogeneic CAR-M may trigger a mild host immune response (16, 36), and its long-term safety needs to be further verified by optimizing HLA matching strategies or gene editing to knock out immunogenic antigens (37, 38).

## 3 Engineering strategy for CAR-M cells and optimization of antitumor mechanisms

### 3.1 Molecular design strategy for CARs

The CAR structure is central to the function of CAR-M-cell therapy, which determines the ability of macrophages to recognize tumors, activate signals, polarize anti-inflammatory phenotypes, and achieve efficacy (39, 40). Its design has undergone an evolutionary process from directly learning from CAR-T-cell technology (16, 41) to exclusive optimization according to the biological characteristics of macrophages (42–44). The current CAR structure aims to improve the survival persistence of CAR-M cells *in vivo*, improve the TME, and further enhance the phagocytosis, tumor killing and antigen presentation ability of macrophages (42).

A CAR is a protein molecule that is artificially designed and synthesized via genetic engineering. Its core function is to give immune cells a new ability to recognize and attack specific targets, such as tumor cells (45). It consists of three major functional components (46): a single-chain variable fragment (scFv) that specifically recognizes target antigens on the surface of tumor cells, a hinge region and a transmembrane domain that links the extracellular and intracellular domains, and an intracellular signaling domain that triggers antitumor effects. The initial CAR-M initially activated only the phagocytic or proinflammatory ability of macrophages toward tumors: the use of CD3ζ or the ITAM motif of the structurally homologous FcϵRI-γ chain to transmit signals effectively initiates antigen-dependent phagocytosis, which is the earliest structural basis to verify the feasibility of CAR-M (16); subsequent introduction of the CD147 intracellular domain of macrophages itself to activate the expression of matrix metalloproteinases (MMPs) (47) or the intracellular domain (TIR) of Toll-like receptors such as TLR4 to mimic pathogen recognition signals (28, 42) initiates only a single basal signal. The basic feasibility of first-generation CAR-M cells was demonstrated in different ways.

Second-generation CAR-Ms can enhance multiple functions, such as phagocytosis, polarization, and proliferation, by integrating dual signals. Zhang et al. (42) realized the dual increase in CAR-M proinflammatory activity and phagocytic intensity by connecting the phagocytosis signal (CD3ζ) and the proinflammatory polarization signal (TIR) in series; Yan et al. (48) expressed Trop2 antigen-targeting second-generation CAR containing CD3ζ and costimulatory signaling molecule 4-1BB in macrophages to achieve the dual effects of phagocytosis enhancement and pro-inflammatory polarization; Ning et al. (43), on the one hand, used the highly expressed FcγRI signal domain of macrophages to enhance phagocytosis, and at the same time introduced α1β1 integrin signal domain to significantly promote the proliferation and survival of CAR-M itself. This “proliferation + phagocytosis” dual enhancement design shows better efficacy and safety than the traditional CD3ζ-CAR.

**Figure 1:**
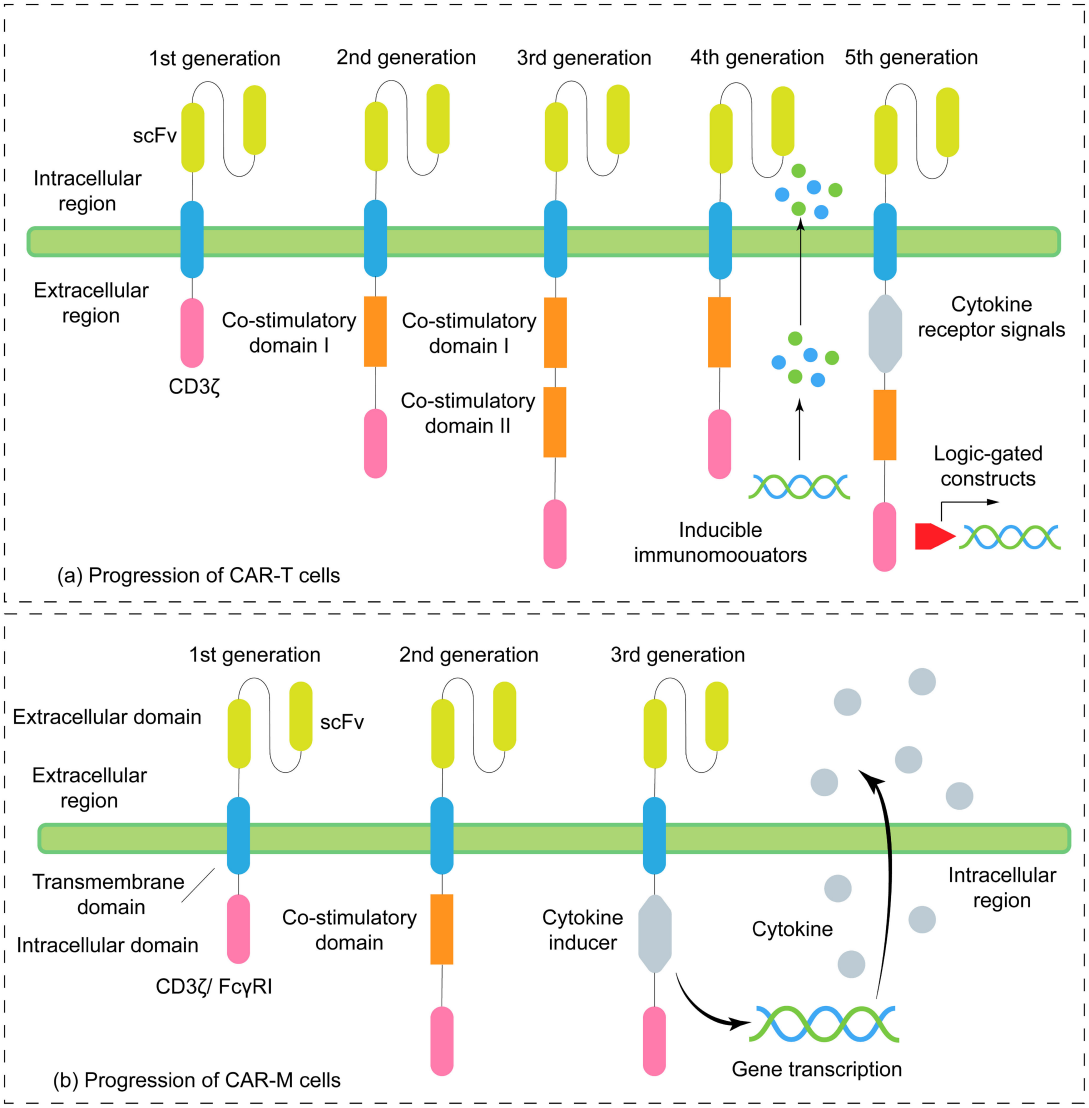
Evolution of CAR structure in CAR-T versus CAR-M cells. **(a)** Progression of CAR-T cells. First-generation CARs were characterized by the inclusion of only the CD3ζ signaling domain and the absence of costimulatory elements. The second generation introduced one costimulatory domain, while the third generation incorporated two. Fourth-generation CAR-T cells are designed to secrete immunomodulatory factors (e.g., IL-12, IL-15) or express checkpoint inhibitors (e.g., anti-PD-1 single-chain variable fragments) to remodel the immunosuppressive tumor microenvironment. Fifth-generation CAR-T cells integrate cytokine receptor signaling domains—such as a truncated IL-2 receptor beta chain (IL-2Rβ) or JAK-STAT module—along with costimulatory domains. These enhancements enable autonomous cytokine-driven proliferation and incorporate logic-gating capabilities. **(b)** Progression of CAR-M cells. First-generation CAR-Ms consisted of a single intracellular domain (e.g. CD3ζ or FcγRI) to trigger phagocytosis. The second generation contains two intracellular domains: one responsible for phagocytosis and the other serving as a costimulatory domain. The third generation further incorporates a cytokine inducer coupling domain.

Third-generation CAR-Ms not only optimize the antitumor function of macrophages but also emphasize the modification of the TME by cells to improve their durability and comprehensive efficacy (49–51). On the basis of second-generation CAR-M, Kang et al. (52) used CD3ζ as the intracellular signal transduction domain and CD28 as the intracellular costimulatory domain and simultaneously expressed IFNγ in tandem, which not only strengthened the proinflammatory phenotype but also reshaped the TME and inhibited immunosuppressive cells (such as Tregs). It also recruits and activates other immune cells, such as T cells, to form synergistic antitumor immunity (**Figure 1**).

Of note, most current CAR-M constructs still utilize the CD3ζ chain derived from conventional CAR-T designs as the core signaling module. This preference stems from the presence of ITAM motifs in CD3ζ, which serve as a well-established signaling hub capable of efficiently initiating lymphocyte activation and macrophage phagocytosis (53). Such a “borrowing” strategy is both rational and efficient during the conceptual validation phase of first-generation CAR-M designs. However, macrophages also possess their own unique signaling receptors, such as MERTK and DAP12 (54, 55). Therefore, the next generation of CAR-M designs should not be confined to the T-cell paradigm, but instead explore signaling domains intrinsic to macrophages themselves. For instance, combining the TIR domain of TLR4 (48) or the intracellular domain of integrins (49) with CD3ζ may allow more precise regulation of macrophage polarization, survival, and phagocytic activity. Future research directions include: 1) screening for the most efficient combinations of intracellular signaling domains to activate macrophage-specific functions, such as M1 polarization, matrix degradation, and antigen presentation; and 2) developing novel synthetic signaling components entirely based on myeloid cell biology, to achieve native-like optimization and precise control of CAR-M function.

### 3.2 Increased solid tumor homing and stromal penetration

The limitations of CAR-T cells in the treatment of solid tumors can be attributed in part to insufficient infiltration due to the loss of chemokine gradients, such as low CXCL9/CXCL10 expression, and physical barriers formed by the extracellular matrix (ECM) (7, 56). In contrast, CAR-Ms can respond to gradient signals of chemokines such as CCL2 and CX3CL1 in the TME by highly expressing chemokine receptors (such as CCR2 and CX3CR1) and achieve efficient directional migration (**Table 1**) (58, 59). In addition, CAR-Ms can secrete MMPs (such as MMP-3, MMP-9, and MMP-14), directly degrade collagen and laminin in the ECM, and break through the physical barrier of tumors (60).

**Table 1:** Tumor infiltration and therapeutic efficiency of CAR-M.

Target antigen	Cell source	Animal model	Methods for evaluating infiltration efficiency	Efficacy outcome	Refs.
HER2	Autologous peripheral blood CD14^+^ monocytes	NSG mice (SKOV3 ovarian cancer)	Immunohistochemistry/ Immunofluorescence	Tumor growth was significantly inhibited, and CAR-M was detected in tumor tissues	(16)
HER2	iPSC	NSG mice (ovarian cancer)	Bioluminescence imaging	It is enriched at the tumor site and inhibits tumor growth	(35)
c-MET	PBMC	Humanized mice (pancreatic cancer)	Flow cytometry/Tissue staining	It was detected in tumor tissue and correlated with tumor shrinkage	(30)
GPC3, FAP	Endogenous macrophages (*in vivo* engineered)	C57BL/6 mice (Hepa1–6 hepatoma)	Immunofluorescence	Deep infiltration into the tumor core degraded ECM and enhanced T-cell infiltration	(57)

A breakthrough study by Zhang et al. (47) provided key evidence for this finding: HER2-targeting CAR-M (CAR-147) significantly upregulated the expression of MMP3, MMP11, MMP13 and MMP14 when cocultured with HER2 + 4T1 tumor cells *in vitro*. In the orthotopically transplanted breast cancer model, the tumor ECM density of the mice in the CAR-147 treatment group was reduced, and the proportion of tumor-infiltrating T cells was increased by 4-fold compared with that in the control group, indicating that CAR-M could synergistically enhance immune cell infiltration through the dual mechanism of “chemochemical degradation” to reshape TME permeability.

Zhang’s team (57) designed a dual-target quadrivalent CAR structure by simultaneously targeting the GPC3 antigen of liver cancer cells and the FAP marker of tumor-associated fibroblasts (CAFs). Immunofluorescence staining revealed that the CAF layer was thinner, the fibrotic area was reduced, and the infiltration depth of CD8^+^ T cells was significantly greater than that in the LNP-Control group. Moreover, the dual-effect design of FAP-CAR-△TGFβRII significantly reduced the p-SMAD2/3 signal in the macrophages of the LNP-GF CAR mRNA group, thereby inhibiting its profibrotic effect. These results indicate that CAR-M cells not only have the ability to penetrate but also cooperate with endogenous immune cells to achieve deep infiltration and long-lasting antitumor effects by systematically removing the fiber barrier and fiber-promoting signals.

### 3.3 Synergistic effects of the multimodal inflammatory response and immunity

CAR-M cells exert anti-tumor effects that extend well beyond direct phagocytic activity. Functioning as a critical nexus between innate and adaptive immunity, CAR-M cells orchestrate multimodal inflammatory responses and promote immune synergy through the secretion of a distinct array of cytokines and chemokines (**Table 2**). As the core immunosuppressive component of the TME, TAMs drive the functional exhaustion of CAR-T cells by secreting immune checkpoint molecules such as IL-10 and TGF-β and expressing PD-L1 (61). The unique advantage of CAR-M cells is that they can remodel the TME through dual mechanisms. On the one hand, CAR-Ms secrete proinflammatory factors such as IL-12 and TNF-α and directly induce the reprogramming of immunosuppressive TAM subsets to a proinflammatory phenotype. On the other hand, it indirectly weakens Treg-mediated immunosuppression by removing the Treg chemokine CCL22 (62). This allows CAR-M cells to not only exert antitumor effects through direct phagocytosis and antigen presentation but also activate systemic immune responses due to their strong proinflammatory properties (63).

**Table 2:** The key effector molecules secreted by CAR-M cells after activation and their functions.

Cytokine/chemokine	Function in CAR-M therapy	Impact on TME	Refs.
IL-12	Drives Th1 immune response, activates CTL and NK cells, inhibits Treg function	Promotes M1 phenotype, enhances T-cell infiltration	(28)
IFN-γ	Activates cytotoxic T cells and NK cells, enhances antigen presentation	Boosts adaptive immunity, skews TAMs toward M1	(16)
TNF-α	Directly induces tumor cell apoptosis, disrupts tumor vasculature	Enhances immune cell infiltration, promotes tumor killing	(27)
CCL5	Recruits CD8^+^ T cells, CD4^+^ Th1 cells, and NK cells to tumor site	Improves T-cell recruitment and tumor immune surveillance	(29)
CXCL9/CXCL10	Chemokines that attract T cells and NK cells, break T-cell exclusion in TME	Enhances immune cell trafficking and infiltration	(29)
MMPs (e.g., MMP-3, -9, -14)	Degrade ECM, promote immune cell infiltration, may release immunomodulatory fragments	Reduces physical barrier, improves T-cell access, remodels stromal architecture	(47)
GM-CSF	Activates monocytes and dendritic cells, enhances antigen presentation	Promotes myeloid cell activation and immune stimulation	(30)
IL-1β	Proinflammatory cytokine, enhances phagocytosis and oxidative stress	Boosts inflammatory response and tumor killing capacity	(44)
NO/ROS	Direct cytotoxic agents, induce oxidative stress in tumor cells	Direct tumor cell killing, enhances phagocytic efficiency	(44)

The study by Klichinsky et al. (16) revealed the molecular regulatory network of CAR-M: transcriptome analysis of anti-CD19 CAR-M (CD3ζ signaling domain) cells revealed that it significantly upregulated the expression of interferon signaling pathway, pattern recognition receptor pathway and proinflammatory phenotype TAM markers (inducible nitric oxide synthase (iNOS) and CD86). *In vitro* coculture experiments further confirmed that CAR-Ms could increase the secretion of IL-12 and downregulate the expression of arginase-1 (Arg1) in proinflammatory TAM subsets.

Zhang’s team (44) blocked the “do not eat me” signal (CD47-SIRPα axis) by silencing the SIRPα gene of CAR-M, increased the secretion of IL-1β by approximately 2–3-fold, and significantly increased the levels of nitric oxide (NO) and reactive oxygen species (ROS). Transcriptome analysis revealed that the expression of NADPH oxidase complex genes (such as NOX2 and RAC1) was upregulated in SIRPα-silenced CAR-Ms, confirming that SIRPα-silenced CAR-Ms enhanced tumor cell death through the oxidative stress pathway.

In the innovative study of Gu et al. (64) CD3ζ/TLR4 dual-signal domain CAR-Ms were constructed through an mRNA-LNP delivery system, which activated the TLR4-Nf-κb pathway in a peritoneal metastasis model, increased the M1/M2 ratio from 1:1 to 3:1, and significantly reduced Treg infiltration (by 10%). Single-cell sequencing further revealed that CAR-M cells could increase the proliferation ability of TCF1+PD-1+ exhausted CD8+ T cells (Tpex) and reconstruct the exhausted phenotype of T cells, which provides a theoretical basis for combined treatment with immune checkpoint inhibitors.

CAR-M cells exert a potent anti-tumor immune cascade by releasing a series of inflammatory cytokines and chemokines, which primarily function through three interconnected mechanisms: 1) Direct reprogramming of the TME: cytokines such as IL-12 and TNF-α polarize neighboring M2-type tumor-associated macrophages (TAMs) toward a pro-inflammatory M1-like phenotype, fostering a self-amplifying anti-tumor environment; 2) Initiation of adaptive immunity: after phagocytosing tumor cells, CAR-M cells cross-present tumor antigens to T cells via MHC-I/II pathways while simultaneously providing critical signals like IL-12 that act as the “third signal” for T cell activation, driving their clonal expansion and differentiation into effector and memory cells; 3) Immune memory formation: this coordinated process ultimately establishes long-term host immune surveillance against tumor neoantigens, constituting a durable *in situ* vaccine effect.

### 3.4 Proliferation and controllability design and safety optimization

At present, the proliferation capacity and controllability of CAR-M cells remain under exploration. A major challenge hindering clinical translation is the limited proliferative ability of terminally differentiated mature tissue macrophages, which leads to insufficient persistence *in vivo* and thus constrains long-term efficacy. This biological limitation highlights the need for a strategic shift in next-generation CAR-M development—from merely mitigating risks to proactively enhancing proliferation capacity and establishing sophisticated safety control systems.

#### 3.4.1 Proactive enhancement of proliferative capacity

CAR-M cells are generally considered to exhibit “transient *in vivo* survival” (65, 66), meaning that their short post-infusion lifespan limits durable antitumor efficacy. To overcome the restricted proliferative capacity of these cells, current research aims to artificially enhance their expansion ability—even toward long-term proliferation—using synthetic biology approaches. For instance, CRISPR activation (CRISPRa) can be applied to upregulate endogenous cell cycle–regulating genes (e.g., c-MYC), or lentiviral transduction can be used to introduce exogenous immortalizing gene combinations (such as Raf and Myc, as validated in iBMDM models) (67, 68). Theoretically, such “long-acting CAR-M” or “immortalized CAR-M” could achieve sustained expansion and residence *in vivo*, enabling continuous tumor immune surveillance and potentially breaking through current therapeutic limitations (**Table 3**).

**Table 3:** Tumor killing efficiency of CAR-M in preclinical studies.

Target antigen	*In vitro* model	*In vitro* killing efficiency	*In vivo* model	Anti-tumor effect *in vivo*	Refs.
HER2	SKOV3 ovarian cancer cells	When E:T ratio was 1:1, the specific phagocytosis rate reached ~60%. At the same time, the secretion of IL-6 and IL-1β increased more than 20-fold.	NSG mice (SKOV3 subcutaneous xenograft tumor)	Tumor burden was reduced by approximately 80% within 30 days after a single infusion; The median survival time of the mice increased from 35 days to >70 days.	(16)
HER2	SKOV3 ovarian cancer cells	At E:T=5:1, the 48-hour cancer cell killing rate was approximately 70% (as determined by a real-time cell analyzer).	NSG mice (SKOV3 subcutaneous xenograft tumor)	Compared with the control group, the treatment group had a 90% reduction in tumor volume on day 28.	(35)
Mesothelin	Pancreatic cancer cells (Capan-2)	The phagocytosis index of SIRPαKO CAR-M at E:T=5:1 was 3.1 times higher than that of the control group. The ROS production was increased by about 2.5 times.	Humanized mice (Pancreatic cancer PDX model)	There was a 4.2-fold increase in CD8+ T cell infiltration in the tumors of the treated mice, and the tumor growth inhibition rate was 75%.	(44)
c-MET	Pancreatic cancer cells (PANC-1)	Under the condition of E:T=10:1, the specific phagocytosis rate was up to 85% within 4 hours. The secretion of GM-CSF increased about 15 times.	C57BL/6 mice (Orthotopic pancreatic cancer model)	In combination with gemcitabine, the median survival time of mice was prolonged from 40 days to 65 days. The incidence of liver metastasis was reduced by 60%.	(30)
GPC3, FAP	Hepa1–6 hepatoma cells	At E:T=10:1, the *in vitro* phagocytic index reached about 11%.	C57BL/6 mice (Hepa1–6 orthotopic liver cancer model)	After a single intravenous injection of LNP-CAR mRNA, about 70% of mice had complete tumor regression and developed a long-term immune memory that was resistant to tumor rechallenge.	(57)
CD19	Raji B-cell lymphoma cells	At E:T=2:1, the killing efficiency was more than 95% at 18 hours (detected by flow cytometry). At the same time, a high level of IL-12p70 (>2 ng/mL) was secreted.	NSG mice (Raji lymphoma model)	The survival rate of CAR-M treated mice was 100% at day 50, while all the control mice died within 35 days.	(42)

#### 3.4.2 Safety control, exhaustion prevention, and dynamic balance strategies

Although terminally differentiated mature tissue macrophages possess limited proliferative capacity, genetic engineering may inadvertently introduce risks associated with uncontrolled activation (16, 65, 69). Sustained CAR signaling could trigger a threefold hazard: excessive inflammation or tissue damage (16, 35, 70), CAR-M cell exhaustion, and functional impairment of tumor-infiltrating T cells (53, 71, 72). More importantly, the sustained secretion of inflammatory factors by CAR-M cells can maintain tumor-infiltrating T cells in a state of prolonged exposure to high antigen levels and co-stimulatory signals. This persistent activation environment may accelerate their differentiation toward a terminally exhausted phenotype, thereby undermining the establishment of long-term adaptive immune memory (73, 74).

Future research should focus on integrating precise control strategies with dynamic equilibrium regulation. On one hand, spatiotemporal precision in CAR-M activity can be achieved by incorporating “suicide switches” (e.g., the iCasp9 system) (75, 76) or designing conditionally activated CARs (such as hypoxia-responsive promoters) (77). CRISPR-Cas9 technology may further be employed to knockout proliferation-related genes such as c-MYC (78, 79) or to restrict their division cycles through epigenetic editing (80, 81). On the other hand, safety designs for next-generation CAR-M should evolve from simple “emergency clearance” toward “dynamic equilibrium regulation.” Specific approaches include developing closed-loop feedback systems in which cytokines (e.g., IL-12) expressed by CAR-M are subject to self-inhibition or linked to metabolite levels (e.g., low arginine) for autonomous regulation (82). Intermittent activation switches can also be constructed to control CAR expression or signaling via exogenous small-molecule drugs, enabling “on-demand activation” and allowing recovery time for the immune system (83–85). Additionally, CAR-M may be engineered to secrete cytokines such as IL-21 or IL-7 under tumor microenvironment regulation to support immune partner cells and delay their functional exhaustion (86, 87).

The core of these strategies lies in shifting from merely pursuing “maximal persistence” to actively designing “optimal therapeutic kinetics.” This approach would allow CAR-M to operate efficiently during tumor clearance phases while remaining silent or being eliminated during phases of potential risk, ultimately achieving an optimal balance between efficacy and safety.

#### 3.4.3 Prospective safety and ethical considerations

While safety measures such as gene editing and suicide switches show promise, their clinical translation requires careful evaluation of potential risks. These include possible off-target effects of CRISPR-Cas9 (88) and immunogenicity associated with exogenous suicide-switch proteins like iCasp9 (76, 89). Ethical concerns must also be addressed, particularly regarding technologies involving embryonic stem cells or heritable genome modifications. Furthermore, the complexity of these engineering strategies is likely to increase production costs, posing challenges to accessibility and future commercialization. Therefore, rigorous safety assessments, thorough ethical review, and cost-reducing innovations must advance in parallel with technological development. In the future, combining *in vivo* delivery systems—such as LNP—based platforms—with modular mRNA engineering may help lower application costs and mitigate ethical concerns, thereby facilitating broader clinical adoption.

## 4 Study of the heterogeneity of cell origins and their suitability for CAR-M technology

### 4.1 Peripheral blood mononuclear cells

As an expanding innovation in CAR-T-cell therapy, the selection and optimization of the cell source of CAR-M-cell technology is the core challenge of clinical translation. Several studies have confirmed that the antitumor activity of PBMC-derived CAR-modified cells is potentially feasible. Xie et al. (90) constructed a second-generation CAR molecule targeting HER2 and reported that PBMC-activated CAR-HER2-T cells significantly inhibited tumor growth and prolonged survival in a tumor-bearing mouse model. Miranda et al. (91) Furthermore, there was no significant difference in the activated phenotype, cytokine secretion or *in vitro* cytotoxicity of CAR-T cells derived from frozen PBMCs and fresh samples, suggesting that frozen PBMCs can be used as a standardized raw material for CAR-T-cell therapy to support global clinical promotion. However, as primary cells, PBMCs have limited proliferation ability *in vitro*, significant phenotypic heterogeneity, and are prone to genetic drift or microbial contamination during culture (92), which severely limits their large-scale clinical application. Therefore, current studies are more inclined to explore other cell sources that can be standardized for expansion.

### 4.2 Human immortalized mononuclear cell line (THP-1)

In 1980, Shigeru TSUCHIY et al. (93) THP-1 cells were isolated and cultured from the blood of a boy with acute monocytic leukemia to clarify the immune function of the monocyte sequence, and an immortalized human monocytic cell line that retains the characteristics of monocytes, such as immune function, was established. As a classic model for the study of monocyte function, the THP-1 cell line has been innovatively applied to the development of CAR-M cells in recent years. This cell line can be induced by a combination of PMA and 1,25-dihydroxyvitamin D3 (VD3) to differentiate into a phenotype characteristic of macrophages, including mitochondrial expansion, upregulation of the antiapoptotic protein Mcl-1, and enhanced resistance to apoptosis (94).

Research team of Yang et al. (95) designed CD147 and CD3ζ intracellular fragments into CARs and screened the HER2-CAR structural design on the basis of CAR-CD3ζ-CD147 through the FLAG tag test of CARs and the antibody-dependent cellular phagocytosis (ADCP) ability test of cells. Moreover, to avoid cytokine storms that can occur late in CAR-M treatment, researchers have stably transfected suicide gene-induced caspase-9 (iCasp9) into THP-1 cells (95) and treated them with different concentrations of AP1903 and different numbers of apoptotic cells. The expression of iCasp9 in THP-1 cells and its ability to rapidly induce THP-1 cell death were detected. Interestingly, the investigators reported that activated CAR-THP-1 cells exhibited an M1 phenotype, which promoted a shift from a suppressive to a proinflammatory immune microenvironment. Owing to its ultrafast average doubling rate of 30–50 hours, low homogeneous genetic background and wide availability, THP-1 cells greatly compensate for the limitations of PBMCs in terms of proliferation and passage ability and the high variability in cell performance caused by diverse genetic backgrounds (96). However, although THP-1-derived monocytes have typical macrophage characteristics, they cannot fully mimic all the properties (97) of macrophages. For example, LPS-stimulated THP-1-derived monocytes produced significantly less IL-6 than did PBMCs and monocytes under the same conditions. However, the secretion of IL-8 in THP-1 cell-conditioned medium was greater than that in THP-1 cell-conditioned medium (98). This finding also suggests that the cell source of CAR-M cells needs more stable and efficient activation in clinical protocols.

### 4.3 Human pluripotent stem cells

To overcome the technical bottleneck of the cell source, hPSCs have become a potential ideal source of CAR-M cells because of their unlimited proliferation potential and genetic editing ability. Zhang et al. (35) developed induced pluripotent stem cells (iPSCs) by reprogramming peripheral blood PBMCs and successfully differentiated them into functional CAR-Ms, which verified the feasibility of hPSC-derived CAR-Ms. Shen et al. (33) developed a monolayer culture system without a feeding layer, combined with the serum-free culture conditions established by Wilgenburg’s team (99), which significantly improved the differentiation efficiency of CD14+CD16lowCD163+ macrophages, laying the foundation for large-scale production. On the basis of the easy editing and immune compatibility of hPSCs, Wang et al. (100) used CRISPR-Cas9 to knock out the ACOD1 gene to increase the reactive oxygen species (ROS) generation ability of hPSC-derived CAR-M (CAR-iMac) and promote its polarization to the M1 type. Furthermore, it has a stronger tumor inhibitory effect in ovarian cancer and pancreatic cancer models. In addition, Lei et al. (42) further optimized the design of CARs and constructed second-generation CAR-Ms with a CD3ζ–TIR dual signal domain, which significantly improved the phagocytic activity and antitumor function of CAR-iMacs. However, the interindividual differences in hPSC differentiation efficiency, phenotypic instability and high production cost are still the main obstacles to their clinical translation.

### 4.4 Future developments in macrophage sources

THP-1 cells, PBMCs and hPSCs have their own advantages and limitations. Although PBMCs are clinically relevant, their expansion ability is insufficient. hPSCs have significant potential, but their differentiation efficiency and cost limit their application. The controllability and function of THP-1 cells are balanced but need to be further optimized to mimic the characteristics of natural macrophages. Future studies need to combine gene editing technology with novel differentiation strategies to overcome existing bottlenecks and promote the clinical translation of CAR-M therapy. Moreover, a study of mouse long-lived macrophages (LT-BMDMs) revealed that mouse bone marrow macrophages can be immortalized via the transduction of functional genes (101). Blasi et al. first named these cells immortalized bone marrow macrophages (iBMDMs) (101). Subsequent studies revealed that iBMDMs exhibited the same functions as primary bone marrow cells in terms of metabolic status, polarization phenotype, and response to external stimuli. Even in a mouse tumor model, it showed stronger antitumor activity than primary macrophages (102) did. A safety evaluation in the latest literature confirmed that iBMDMs could survive for 35 days *in vitro* and maintain vigorous proliferation activity for 4 weeks. The body has a life span of three weeks (102). Compared with those of primary BMDMs, such proliferation activity and survival ability are great breakthroughs, and the safety hazard caused by immortalization is overcome; thus, the authors named these cells long-lived macrophages (LT-BMDMs). These mouse LT-BMDMs have proliferative activity, editing ability, and powerful antitumor functions, suggesting the possibility of a new cell source for macrophage therapy in the future. Is it possible to induce and construct long-lived macrophages (hLT-BMDMs) from human primary bone marrow macrophages? Min Peng successfully screened the key regulatory genes of immortalized CAR-T cells via CRISPR-Screen technology. Although its safety and clinical application still need to be further verified and evaluated, it also provides a new idea for the construction of hLT-BMDMs.

## 5 Discussion: the bottleneck of CAR-M-cell therapy

### 5.1 TAM heterogeneity and the domestication of the immunosuppressive tumor microenvironment

However, preclinical studies and early clinical data suggest that CAR-M cells exhibit unique advantages over CAR-T cells in terms of phagocytosis, antigen presentation, and remodeling of the TME (13, 103, 104). These features make CAR-M cells promising immunotherapy agents for T-cell-refractory solid tumors. However, at the same time, the functional and spatial heterogeneity of TAMs, as well as the “domestication-depletion” effect of the TME, pose challenges for the further clinical translation of CAR-M cells.

Recent advances in spatial transcriptomics and single-cell technologies have further revealed unprecedented functional and spatial heterogeneity within TAM compartments (105, 106). Instead of having a uniform distribution, TAMs are organized into specific “ecotypes,” with distinct isoforms distributed in necrotic zones, hypoxic zones, invasion fronts, and perivascular spaces, each exhibiting a unique transcriptional program and role in immune regulation (67, 107, 108). This complex TME dynamic presents a new set of challenges for CAR-M therapy. First, different organ-specific microenvironments (e.g., liver, lung, and brain) cultivate resident macrophages in unique ways, which means that CAR-M cells may need to be tailored for different organ situations to overcome the local immunosuppressive network (68, 108–111). Second, owing to the regional specificity of different tumor sites in the same organ (such as the tumor core hypoxic zone, invasive marginal zone, and tertiary lymphatic structure), resident TAMs can also show different functional phenotypes (105, 112, 113), which means that different CAR-M cells may also need to be customized for different specific regions of tumors in the same organ. Finally, the long-term functional stability of CAR-M cells is under constant threat from highly specialized local signals, such as IL-10 and TGF-β in the TME, as well as from tumor-associated fibroblasts and the extracellular matrix, which may reprogram the phenotype of CAR-M cells and eliminate their antitumor activity (114, 115).

Overcoming these spatial and environmental barriers will require next-generation engineering strategies. Owing to their high permeability and retention effect on solid tumors (116), the recent *in vivo* editing of macrophages by LNPS may provide new ideas for solving the problems of different “ecotypes” and functional exhaustion of TAMs (117–119). On the basis of the dual delivery system of LNP-mRNA, Yang et al. (120) successfully constructed a bifunctional CAR-M that simultaneously targeted GPC3 and CD24-Siglec-G inhibitory signals. Targeted delivery to HCC-associated macrophages is achieved by the adsorption of specific plasma proteins. *In vitro*, CAR-M coexpressing GPC3-CAR and Siglec-GΔITIMs significantly increased the phagocytic capacity of GPC3^+^ Hepa1–6 cells by approximately 10-fold. Moreover, the expression of Siglec-GΔITIM can block the immunosuppressive signal mediated by CD24 and further enhance the antitumor function of CAR-M cells. This effectively avoids the heterogeneity problem of TAMs caused by dynamic changes in the TME, and endogenous CAR-M cells may show stronger natural tolerance to the local TME due to their “local advantage” (121–123).

Zhang et al. (57) used the LNP-mRNA system to edit macrophages *in situ in vivo* and constructed a quadrivalent CAR-M with GPC3-CAR-Super IL-2 and FAP-CAR-△TGFβRII. The experimental data revealed that after intravenous injection of LNP-GF CAR mRNA, approximately 70% of DID-labeled LNPs were taken up by liver macrophages, confirming its efficient targeting to endogenous macrophages. *In vitro* experiments further revealed that CAR-M increased the phagocytosis index of GPC3^+^ Hepa1–6 cells to approximately 11% and almost completely inhibited tumor growth at a high E/T ratio (10:1). More importantly, despite their transient expression, CAR-Ms successfully induced long-term T-cell immune memory through antigen presentation and local interactions with Super IL-2, which not only eliminated GPC3-positive tumors but also neutralized antigen-negative tumors through antigen diffusion, thus effectively preventing immunosuppressive reprogramming of the TME and tumor recurrence. This strategy provides a dual mechanism of immediate killing and long-term protection for the treatment of solid tumors, highlighting the potential of *in vivo* editing of CAR-M cells to overcome tumor heterogeneity and dynamic changes in the TME.

The LNP-mRNA *in vivo* delivery strategy offers a novel approach to overcoming TAM heterogeneity and reprogramming the immunosuppressive TME. By directly editing endogenous macrophages *in situ* across different TME niches, the resulting CAR-M cells—already present within the TME and pre-adapted to local conditions such as hypoxic or perivascular regions—naturally circumvent the “acclimatization” issues often faced by *in vitro*-modified CAR-M cells during homing and local adaptation. This “*in situ* transformation” strategy allows CAR-M to cover various functional regions inside the tumor, thereby addressing the challenge posed by spatial and functional heterogeneity of TAMs in achieving uniform therapeutic efficacy.

### 5.2 Tumor antigen heterogeneity

Conventional CAR cell therapy, which operates on the principle of specific “antigen–antibody” binding, has long faced the challenge of tumor antigen heterogeneity. Its high specificity often leads to immune escape due to antigen loss or downregulation, while on-target/off-tumor toxicity frequently forces the selection of heterogenous target antigens—a persistent therapeutic dilemma (124–126).

**Figure 2:**
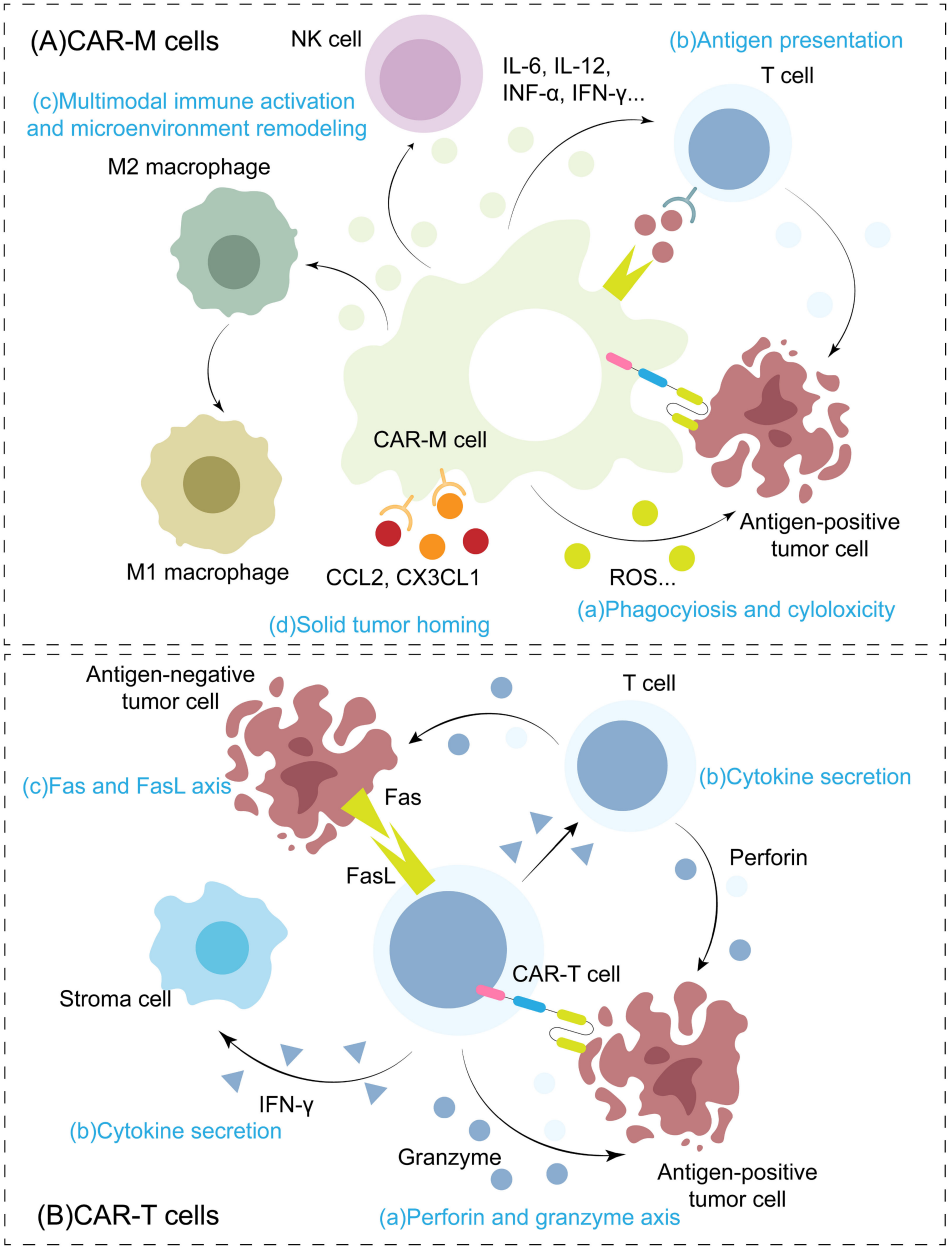
Differences in anti-tumor mechanisms between CAR-T cells and CAR-M. **(A)** Anti-tumor mechanisms of CAR-M: **(a)** Direct phagocytosis and cytotoxicity: CAR-M directly engulfs tumor cells, accompanied by the secretion of reactive oxygen species (ROS) and other cytotoxic factors. **(b)** Antigen presentation and T-cell activation: promotion of T-cell activation and proliferation through efficient antigen presentation. **(c)** Remodeling of the immune microenvironment: the secretion of proinflammatory cytokines such as IL-6, IL-12, TNF-α, IFN-γ and other immune cells can activate T cells, NK cells and other immune cells, and induce the polarization of M2 macrophages to M1 macrophages, and finally reshape the tumor immune microenvironment. **(d)** Efficient directional migration: with the help of highly expressed chemokine receptors (such as CCR2 and CX3CR1), it responds to the concentration gradient of chemokines such as CCL2 and CX3CL1 in the tumor microenvironment to achieve efficient directional invasion. **(B)** Anti-tumor mechanisms of CAR-T cells: **(a)** Direct cytotoxicity: by releasing perforin and granzyme B, CAR-T cells directly lyse tumor cells expressing target antigens. **(b)** Immune activation and bystander effect: it promotes the activation and proliferation of bystander T lymphocytes in itself and the tumor microenvironment by secreting cytokines such as IFN-γ.(c) Antigen-independent killing: tumor heterogeneity can be overcome by inducing apoptosis of tumor cells that do not express target antigens through the Fas/FasL signaling pathway.

CAR-M therapy, however, built on a unique macrophage chassis, has reshaped this landscape. Unlike CAR-T cells, which rapidly induce target cell apoptosis via perforin and granzyme B within hours (127, 128), the core mechanism of CAR-M revolves around the phagosome–lysosome degradation pathway (16). Upon binding of the CAR single-chain variable fragment (scFv) to a tumor antigen, intracellular signaling triggers actin cytoskeleton reorganization, leading to the formation of phagocytic cups that engulf the target cell. The internalized target is enclosed within a phagosome (35), which subsequently fuses with lysosomes. Within the acidic environment of the phagolysosome, tumor cells are thoroughly degraded by hydrolases, reactive oxygen species, and reactive nitrogen species (129). This “root-and-branch” elimination not only physically eradicates cancer cells but also generates abundant tumor antigen fragments. These fragments serve as ideal substrates for efficient cross-presentation to T cells, thereby potently activating the adaptive immune system and establishing a long-term “*in situ* vaccine” effect (**Figure 2**).

In a key study, Klichinsky et al. (16) demonstrated that CAR-M could phagocytose tumor cells expressing NY-ESO-1 antigen but lacking the cognate HLA-A2 molecule. When these CAR-M cells were co-cultured with NY-ESO-1–specific T cells, they robustly activated T cell responses, as indicated by significant upregulation of CD69 and high levels of IFN-γ secretion. This finding confirmed that CAR-M processes phagocytosed tumor antigens and cross-presents them via the MHC-I pathway to CD8+ T cells, effectively initiating tumor-specific T cell immunity. This process lies at the heart of the “*in situ* vaccine” effect: local immune reprogramming within the tumor stimulates systemic adaptive immunity, establishing durable immune surveillance and protection.

Further reinforcing this concept, Zhang et al. (57) developed a quadravalent CAR-M targeting GPC3 and FAP and elucidated its role as an “*in situ* vaccine.” Mice cured by this therapy completely rejected subsequent challenges with GPC3-negative tumors—a protective effect abolished upon T cell depletion, indicating dependence on long-term T cell memory. Using an antigen spreading model, the authors showed that after engulfing GPC3^+^ tumor cells, CAR-M cross-presented the internal model antigen OVA to antigen-specific CD8^+^ T cells, thereby triggering T cell responses against non-CAR target antigens. This study confirmed that, although CAR-M persistence may be transient, its ability to mediate antigen cross-presentation and local secretion of super IL-12 can establish a relatively sustained “*in situ* vaccine” effect capable of overcoming antigen heterogeneity—offering a promising strategy for preventing tumor recurrence.

Through this unique mechanism of stimulating systemic antitumor immunity, CAR-M has the potential to fundamentally dismantle the heterogeneous defense network of tumors, thereby enabling more effective control of solid cancers.

### 5.3 Target-specific challenges and risks of on-target/off-tumor toxicity

While CAR-M therapy holds the theoretical potential to overcome intratumoral antigenic heterogeneity through an “*in situ* vaccine” effect, its therapeutic window fundamentally relies on the specific recognition of target antigens by the CAR structure—a principle that shares the same risk of “on-target/off-tumor” toxicity observed in CAR-T therapy (125). This risk stems from the fact that many desirable tumor-associated antigens (TAAs) are also expressed, even if at low levels or in a temporally restricted manner, in normal tissues (126). Clinical experience with CAR-T cells has clearly demonstrated this challenge: for instance, ERBB2-targeted CAR-T cells induced fatal pulmonary toxicity due to low-level target expression in the lung (130), while CAIX-directed CAR-T cells caused reversible liver injury by attacking bile duct epithelial cell (131). As a therapeutic approach built on the same targeting logic, researchers in the CAR-M field have explicitly acknowledged from the outset that CAR-M cells targeting TAAs may similarly inflict “on-target, off-tumor” toxicity on normal tissues expressing the same antigen (16).

It should be emphasized, however, that the off-tumor toxicity profile of CAR-M may differ from that of CAR-T. Owing to the potent phagocytic capacity, robust inflammatory secretion, and tissue-resident properties of macrophages, their attack on normal tissues could lead to more widespread and sustained inflammatory damage, potentially driving chronic fibrosis (16, 70). This concern is grounded in solid pathophysiological rationale: persistently activated macrophages are established key drivers of tissue fibrosis, promoting excessive extracellular matrix deposition (132). Furthermore, under conditions of immune hyperactivation, macrophages have been shown to serve as primary effector cells mediating extensive inflammatory tissue injury and subsequent fibrotic remodeling (133). For example, HER2-directed CAR-M cells might not only target HER2^+^ tumor cells but also affect normal tissues such as the heart where cardiomyocytes exhibit low HER2 expression, lungs, or skin, potentially causing myocarditis, pneumonitis, or dermatitis (130, 134). The injury pattern in such cases may skew more toward disordered tissue remodeling and chronic fibrosis rather than acute necrosis. Additionally, the strong antigen-presentation and immune-activating functions of CAR-M represent a double-edged sword. Theoretically, normal tissue debris phagocytosed by CAR-M could be processed and presented to T cells, thereby activating responses against self-antigens and potentially triggering autoimmune-like adverse effects. While direct evidence remains to be accumulated, the secondary autoimmune phenomena already observed in CAR-T therapy following on-target damage to normal tissues (135) underscore the need for high vigilance regarding this risk.

To strictly confine CAR-M activity in time and space, next-generation engineering strategies are focusing on novel synthetic biology designs and logic-gate optimizations. These include, for example, TME-responsive logic-gate systems or tissue-restricted promoters engineered to silence CAR expression in specific normal tissues (124). Preclinical validation can be enhanced through humanized mouse models that simulate the systemic distribution and organ-specific responses of CAR-M in a humanized immune context (136). Furthermore, patient-derived organoid co-culture systems can directly assess the potential aggressiveness of CAR-M against normal human tissues expressing the target antigen in a near-physiological setting, providing indispensable personalized safety predictions (137). In summary, advancing CAR-M toward successful clinical translation will require a dual focus: maximizing anti-tumor potency while proactively mitigating off-tumor risks through refined and intelligent engineering design.

### 5.4 Challenges in manufacturing and phenotype standardization

The clinical translation of CAR-M therapy depends not only on innovative receptor design but also faces a more pronounced bottleneck than CAR-T: the fundamental tension between the high intrinsic plasticity of macrophages and the batch-to-batch consistency required for clinical-grade manufacturing (138, 139). Macrophage polarization is not a simple M1/M2 dichotomy but a continuous functional spectrum finely tuned by microenvironmental signals. For instance, GM-CSF primarily drives M1-like polarization with high expression of IL-12 and inducible iNOS, while M-CSF promotes an M2-like phenotype characterized by elevated IL-10 and arginase 1 (139). Single-cell sequencing further reveals that even monocytes from the same donor, cultured under standardized conditions, can differentiate into transcriptionally and functionally heterogeneous subsets, with phagocytic capacity varying by up to threefold across subpopulations (140). This inherent plasticity makes the standardization of CAR-M phenotype and function a multidimensional engineering challenge.

Current production processes are highly sensitive to microenvironmental variables. First, minor fluctuations in medium composition can have significant functional consequences. For example, exosomal miRNAs (such as miR-21) present in different serum lots can modulate macrophage activity via the TLR4/NF-κB pathway, directly affecting inflammatory responsiveness (141). Second, physicochemical parameters play a decisive role. Variations in oxygen tension (5% vs. 21% O_2_) can reprogram the metabolome of M2 macrophages, altering levels of TCA cycle intermediates, amino acids, and fatty acids (142). Similarly, substrate stiffness (1 kPa vs. 50 kPa) profoundly influences polarization and function through mechanisms such as YAP/TAZ pathway activation (143); Furthermore, conventional surface-marker-based assays (CD86/CD206) are insufficient to capture functional diversity. PD-L1^+^ macrophages, for example, exhibit distinct immunosuppressive activity in the tumor microenvironment, requiring integrated multi-omics profiling—transcriptomic, metabolomic, and epigenomic—for accurate characterization (144, 145); Finally, source-dependent heterogeneity complicates standardization. Even under identical culture conditions, CD14^+^ monocytes from healthy donors display significant functional variation (146). While iPSC-derived macrophages offer a theoretically uniform starting cell source, their differentiation—especially from hematopoietic progenitors to monocytes—often suffers from low efficiency (<30%) and poor synchrony, leading to heterogeneous final products (147).

To overcome these barriers, emerging strategies integrate process control, cellular engineering, and intelligent monitoring. Innovations in bioprocessing include dynamic perfusion culture, which enhances M1 phenotype stability by 27% compared to static systems (148), and chemically defined media supplemented with metabolites such as butyrate (0.5 mM) to maintain M2 phenotype for over seven days (149). A more transformative approach lies in synthetic biology-based “hardwiring” of cell function. For instance, overexpression of the master transcription factor MAFB in the THP-1 cell line increased expression of the M2 marker CD163 eightfold, indicating that epigenetic engineering can enable phenotypic locking (149).

Looking forward, standardized CAR-M manufacturing must converge with intelligent, precision-platform technologies. Priority directions include microfluidic single-cell culture systems for real-time, non-invasive tracking of phenotypic drift; machine learning-assisted medium optimization leveraging metabolomic and other omics datasets to predict ideal cytokine and nutrient combinations; and improved targeted cryopreservation methods to minimize post-thaw functional loss. In summary, achieving phenotype standardization in CAR-M necessitates interdisciplinary integration across biology, engineering, and data science. Only through the convergence of precision bioprocessing, deep cellular engineering, and intelligent quality control can this promising cell therapy evolve into a reliable, consistent, and clinically deployable “off-the-shelf” anticancer agent.

## 6 New engineering strategy and logic-gated optimization for CAR-M

**Figure 3:**
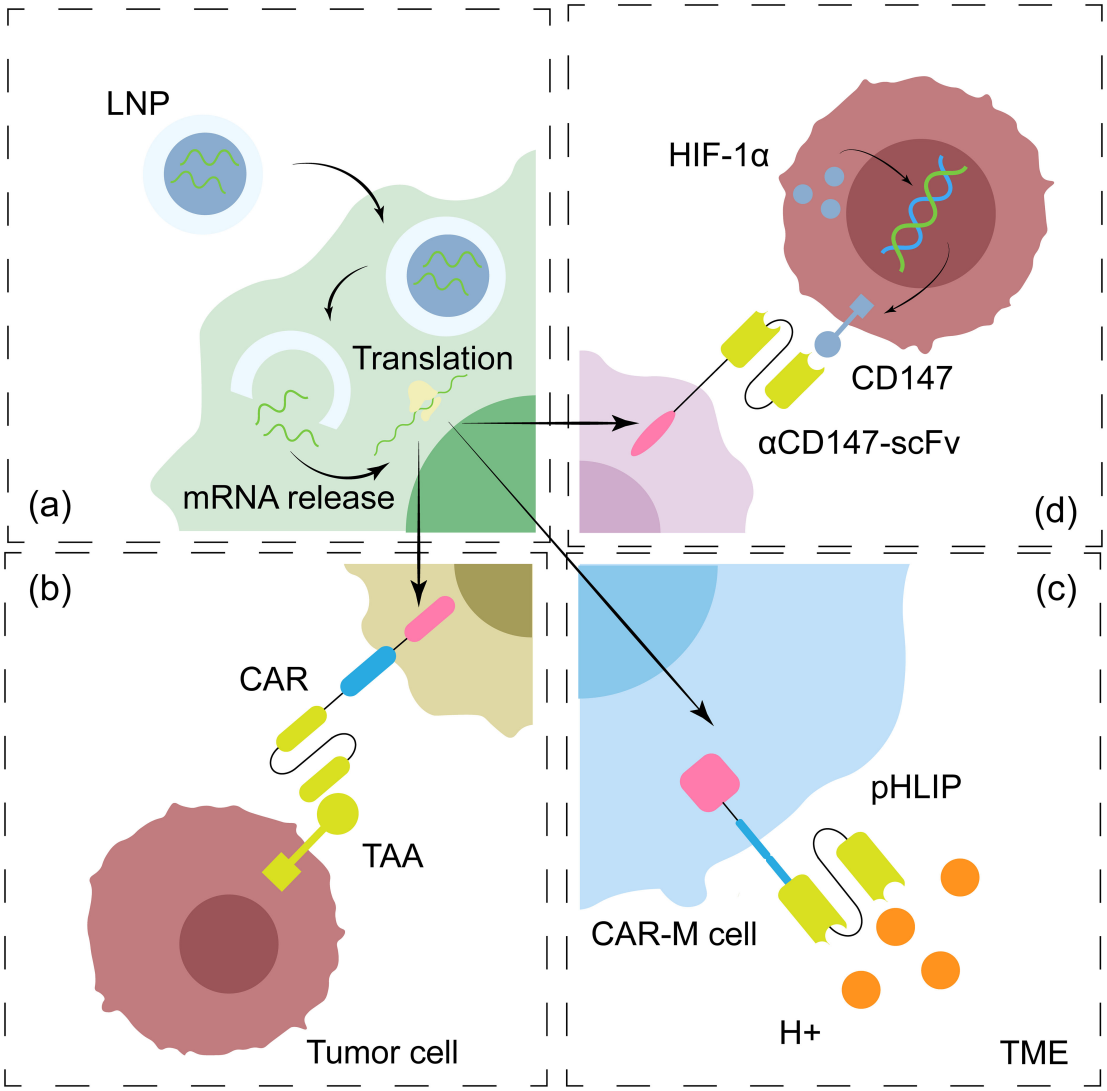
New engineering strategy and logic-gated optimization for CAR-M. **(a)** The *in vivo in situ* generation of CAR-M using LNP technology has emerged as a pivotal strategy for optimizing and advancing the clinical translation of this therapeutic approach. **(b)** Conventional CAR designs typically target a single tumor marker; however, prolonged exposure to tumor microenvironment (TME) pressures can readily induce clonal escape mutations in CAR-M, leading to a progressive loss of targeting efficacy. The distinct physicochemical properties of the TME provide a molecular foundation for developing novel CAR logic-gated systems: **(c)** In the acidic TME, incorporating pHLIP into the hinge or transmembrane domain of CAR may enable controlled activation of CAR-M specifically under acidic conditions; **(d)** whereas in hypoxic regions, leveraging CD147—a protein highly upregulated via HIF-1α stabilization—allows for specific CAR-M activation exclusively within hypoxic microenvironments.

By integrating the innate tumor-homing ability, antigen-presenting function and engineered killing module of macrophages, CAR-M therapy achieves a dual breakthrough in the infiltration of the solid tumor TME and immune remodeling, which represents an innovation in the tumor immunotherapy paradigm (**Table 4**). However, the clinical translation of this technology still faces multiple challenges: immune remodeling may trigger systemic cytokine release syndrome (CRS) (153), and long-term TME pressure easily induces clonal escape mutations in CAR-M cells, leading to attenuated targeting (60, 153). Moreover, the conventional manufacturing process remains complex, involving isolation, genetic modification, and expansion of cells *in vitro* before reinfusion—a procedure that is both costly and time-consuming. Therefore, moving beyond the traditional framework of *in vitro* CAR design, generating or editing CAR-M cells *in vivo*, and developing responsive logic-gate systems tailored to the TME have emerged as pivotal directions for optimizing CAR-M functionality and enabling its clinical translation (**Figure 3**).

**Table 4:** Advances in CAR-M for cancer research.

Target antigen	Intracellular domain	Target tumor or cell	CAR delivery method	Cell source	Refs.
HER2	CD3ζ	HER2-overexpressing solid tumors	Adenoviral transduction	Autologous peripheral blood CD14^+^ monocytes	(16)
HER2	CD3ζ	HER2-overexpressing solid tumors	Adenoviral transduction	BMDM	(23)
c-MET	FcRγ + CD19 PI3K	Pancreatic cancer (BxPC-3, PANC1, AsPC1)	Lentiviral transduction	THP-1, hMDM	(30)
CD19	CD3ζ	K562-CD19 leukemia cells, OVCAR3/ASPC1 ovarian/pancreatic cancer cells, HO8910 ovarian cancer cells	Lentiviral transduction	PBMCs	(35)
HER2	CD3ζ	SKOV3 (ovarian cancer)	Adenoviral transduction	Primary human CD14+ monocytes	(16)
GPC3	CD3ζ+TIR	HepG2 (hepatocellular carcinoma)	Lentiviral transduction	iMACs	(42)
HER2	α1β1 integrin + FcγRI	HER2-positive solid tumors (SKOV3, HER2 + 4T1)	Lentiviral transduction	THP-1	(43)
HER2	CD147	HER2-4T1 breast tumor	Lentiviral transduction	Raw264.7 macrophage	(47)
Trop2	CD28 + CD3ζ	Trop2-overexpressing solid tumors (breast cancer, pancreatic cancer)	mRNA-LNP	Endogenous macrophages (*in vivo* engineered)	(48)
ALK	CD3ζ	Neuro-2a (ALK^+^ neuroblastoma)	MPEI/piggyBac nanocomplex	M2-to-M1 reprogrammed macrophage	(52)
GPC3, FAP	CD3ζ+ Super IL-2+△TGFβRII	Hepatocellular carcinoma (HCC), Cancer-associated fibroblasts (CAFs)	mRNA-LNP	Endogenous macrophages (*in vivo* engineered)	(57)
HER2, CD47	CD3ζ+CD40+TLR4	Peritoneal metastasis (CT26, PAN02, SKOV3 models)	mRNA-LNP	Peritoneal macrophages (*in situ* programming)	(150)
HER2	CD3ζ + CD147	Ovarian cancer (SKOV3), breast cancer (MDA-MB-453), gastric cancer (NCI-N87)	Lentiviral transduction	THP-1	(95)
MSLN	CD3ζ	Ovarian cancer (HO-8910),pancreatic cancer (ASPC-1)	Lentiviral transduction	iPSC	(100)
HER2	CD3ζ	HER2-overexpressing solid tumors(breast, gastroesophageal, salivary duct)	Adenoviral transduction	Autologous peripheral blood CD14^+^ monocytes	(103)
HER2	FcϵRIγ	Peritoneal carcinomatosis	Lentiviral transduction	Human peritoneal macrophages	(151)
c-MET	FcRγ+ CD19 PI3K + CD3ζ	Pancreatic ductal adenocarcinoma	Lentiviral transduction	THP-1, hMDM, BMDM	(30)
MSLN	CD3ζ	Ovarian cancer, primary peritoneal cancer, fallopian tube cancer, peritoneal mesothelioma	mRNA-based electroporation (CARMA platform)	PBMCs	(152)
GPC3	CD3ζ	Hepatocellular carcinoma (HCC)	mRNA-LNP	Liver macrophages (*in situ* programming)	(120)

Breaking through the traditional *in vitro* preparation framework and generating CAR-M cells *in vivo* has emerged as a key strategy to advance the clinical translation of this therapy. Among various approaches, lipid nanoparticle (LNP)-based delivery systems show particular promise. Billingsley et al. (154) systematically validated the potential of LNPs as efficient delivery vehicles by constructing a library of 24 ionizable lipids and identifying the optimal formulation, C14-4, for delivering mRNA into primary human T cells and monocytes/macrophages, thereby inducing the expression of functional CD19-CAR protein. Building on this foundation, Zhou et al. (48) achieved a critical transition from a general platform to CAR-M-specific therapy. They ingeniously designed LNPs loaded with Trop2-CAR plasmid DNA and successfully reprogrammed macrophages into CAR-M cells *in situ* in tumor-bearing mice through either intravenous or intratumoral injection. Together, these studies outline a clear developmental trajectory for *in vivo* CAR-M generation using LNP technology. However, several challenges remain in current LNP research, including how to prevent off-target cell modification due to LNP leakage (48, 155, 156) and how to enhance the targeting efficiency toward specific myeloid cell subsets. Beyond LNPs, emerging strategies involve implantable biological scaffolds loaded with chemokines, cytokines, or genetic materials that can recruit host immune cells and “educate” them *in situ* to become CAR-M cells, potentially enabling long-term CAR expression *in vivo*. For instance, Gao’s team (157) successfully generated CAR-M cells in the brain tumor region through local injection of gene nanocapsules, while Liu’s group (158) developed multifunctional nanopolymers that simultaneously enable CAR expression and macrophage phenotype modulation, demonstrating the precision and synergistic potential of *in vivo* editing strategies. Nevertheless, several aspects of these approaches require further optimization, including the renewal pathway after loaded materials are depleted (159), the biocompatibility of carrier materials, and the controllability of release kinetics (160). These *in vivo* strategies collectively offer innovative solutions to challenges related to cell sources, heterogeneity, and manufacturing costs in the clinical development of cellular immunotherapies.

While pursuing *in situ* editing strategies, researchers are also moving beyond the conventional “antigen–antibody” CAR design paradigm by leveraging distinctive features of the tumor metabolic microenvironment to achieve breakthroughs in targeted immunotherapy. The unique physical and chemical properties of the TME provide a molecular basis for the design of new CAR logic gates. In the hypoxic microenvironment, prolyl hydroxylase (PHD) activity is inhibited, leading to the stable accumulation of hypoxia-inducible factor-1α (HIF-1α) and the activation of downstream prosurvival signaling pathways (161). Notably, this pathway upregulates several tumor-specific or highly expressed surface proteins, such as carbonic anhydrase IX and CD147 (162–164). This observation led us to hypothesize that a CAR could be engineered to target HIF-1α-induced surface antigens. By leveraging this strategy, CAR-M cells can utilize their scFv domain to accurately identify and eradicate tumor cells actively surviving in the hypoxic microenvironment, thus conferring highly specific targeting and minimized off-target toxicity; pH-low insertion peptide (pHLIP) is a 35-amino acid pH-sensitive peptide that enables the transport of conjugated nanocarriers into cells to facilitate phiven active targeting (165). Tang et al. (166) coupled pHLIP to the nanodrug delivery system, which significantly improved the efficiency of drug accumulation at the tumor site. Therefore, the integration of pHLIP into the hinge region or transmembrane domain of CAR is expected to achieve the controlled activation of CAR-M in the acidic TME and reduce the risk of CRS caused by nonspecific immune activation. The introduction of artificial intelligence (AI) technology has further expanded the innovation dimension of CAR design. Through high-throughput data mining, deep learning algorithms can predict the spatial conformation and affinity of the CAR domain and optimize the synergistic effect between the antigen recognition module and the costimulatory signal domain.

Notably, CAR-M therapy demonstrates inherent advantages over conventional CAR-T in terms of CRS safety. Studies indicate that CAR-NK cells exhibit a more favorable safety profile with regard to CRS and neurotoxicity; similarly, CAR-M, as an innate immune cell-based platform, has also shown promising safety outcomes (167). Furthermore, logic-gating designs help mitigate CRS risk by raising the activation threshold of CAR-M. Since activation requires the simultaneous fulfillment of multiple conditions, logic-gated CAR-M remain inactive in normal tissues, thereby avoiding unintended immune activation and cytokine release (168, 169). Additionally, preventive strategies against CRS align with and complement the logic-gating concept. For instance, neutralization or genetic inactivation of granulocyte-macrophage colony-stimulating factor (GM-CSF) has been shown to significantly reduce the secretion of monocyte-dependent CRS biomarkers—including MCP-1, IL-6, and IL-8—without compromising the antitumor efficacy of CAR-T cells (170). Similar approaches could be applied to CAR-M therapy to further diminish the risk of CRS.

At present, several clinical trials of CAR-M cells have entered the early clinical validation stage, and the preliminary results support its further development (**Table 5**). Recently, the world’s first CAR-M therapy (CT-0508, NCT04660929) (103) used the chimeric adenovirus vector AD5F35 to modify macrophages and target the HER2 antigen. Phase I clinical trial data revealed that the number of tumor-infiltrating lymphocytes (TILs) increased approximately 2.1-fold after infusion. The expansion and activation of T-cell clones were significantly enhanced, and no CRS events above grade 3 were observed, which initially verified the safety and feasibility of CAR-M cells and their great advantages for future clinical promotion. In addition, intraperitoneal infusion of autologous HER2-directed CAR-M is planned to evaluate local antitumor effects in patients with HER2-positive advanced gastric cancer and peritoneal metastasis (NCT06224738) (151). Another study (NCT05007379) aimed to systematically evaluate the killing activity of CAR-M cells in breast cancer with different HER2 expression levels by using patient-derived organoid models to provide a basis for individualized treatment.

**Table 5:** Progress in CAR-M related clinical trials.

Clinical trial ID	Phase	CAR-M product	Study status	Target antigen	Conditions
NCT06224738	I	NA	Not yet recruiting	HER2	Gastric cancer
NCT05164666	I	TAK-103	Terminated	MSLN	Solid tumors
NCT05007379	I	NA	Unknown	HER2	Breast cancer
NCT04660929	I	CT-0508	Active not recruiting	HER2	HER2-positive solid tumors
NCT04405778	I	TAK-102	Terminated	GPC3	GPC3-positive solid tumors
NCT03608618	I	MCY-M11	Terminated	MSLN	Ovarian cancer and peritoneal mesothelioma
NA	Preclinical research	CAR-M-c-MET	Terminated	c-MET	Pancreatic cancer

In terms of target expansion, a recent preclinical study on pancreatic cancer revealed the great potential of c-MeT-targeted CAR-M (CAR-m-c-Met) (30). Through bioinformatics and tissue microarray analysis, they confirmed that c-MET was highly expressed in pancreatic cancer tissues and was associated with poor prognosis. *In vitro* and orthotopic mouse pancreatic cancer model experiments revealed that CAR-M-c-MET could specifically recognize and efficiently phagocytose c-MeT-positive tumor cells and secrete GM-CSF and other immune activators to reshape the tumor microenvironment. In addition, no obvious toxicity or side effects were observed in the mouse model, providing solid data support for future clinical research.

Although the field of CAR-M has broad prospects, its development process is also accompanied by strategy adjustment and experience accumulation. For example, earlier studies, such as MY-M11 (NCT03608618), which targets mesothelin (MSLN), and TAK-103 (NCT05164666), developed by Takeda, have been discontinued. An in-depth analysis of these cases can provide a valuable reference for follow-up research. The strategy of MY-M11 using peripheral blood mononuclear cells (PBMCs) to generate CAR-Ms *in vitro* and undergo intraperitoneal infusion is safe in the early stage but may not yield sufficient therapeutic advantages because of insufficient antitumor efficacy or limited cell persistence (152). The termination of these early explorations reflects the challenges of CAR-M technology in terms of cell preparation, *in vivo* persistence, and target selection. It also prompts the field to focus resources on more promising candidates, such as CT-0508, and motivates the development of next-generation engineering strategies (e.g., logic gating and *in situ* generation).

## 7 Conclusion and perspectives

In the field of cellular immunotherapy, CAR-M, an emerging treatment method, has gradually shown great potential in tumor treatment. By integrating the innate immune characteristics of macrophages with synthetic biological modification strategies, the immunosuppressive TME can be reprogrammed, which provides a new paradigm for the treatment of solid tumors. Current studies have revealed multiple antitumor mechanisms involving the secretion of proinflammatory factors (such as TNF-α and IL-12), the induction of M1 polarization, and the enhancement of T-cell cross-presentation. However, the heterogeneity of cell sources, insufficient persistence *in vivo* and bottlenecks associated with large-scale production still need to be overcome.

Future research on CAR-M cells is expected to focus on multiple dimensions. 1) Multimodal gene editing technology: CRISPR-Cas9 and base editing technology are used to target the metabolic pathway of CAR-M or integrate the suicide switch (iCasp9) to balance efficacy and safety; 2) microenvironment-responsive intelligent design: develop light-controlled, tissue-responsive, pH- or oxygen concentration-dependent CAR logic gate systems to achieve space-time specific activation; 3) interdisciplinary technology integration: combine single-cell transcriptome sequencing and an AI-driven CAR optimization platform to analyze the dynamic functional phenotype of CAR-M and predict treatment response; 4) clinical translation verification: multicenter clinical trials were conducted to evaluate the universality of CAR-M in heterogeneous tumors, and a standardized production quality control system was established. With the coordinated development of synthetic biology, computational immunology and precision medicine, CAR-Ms are expected to overcome the barriers associated with solid tumor treatment and become the core pillar of the next generation of tumor immunotherapy.

## Glossary

ACOD1: Aconitate Decarboxylase 1
ADCP: Antibody-Dependent Cellular Phagocytosis
Ad5F35: Adenovirus 5-Fiber 35
AI: Artificial Intelligence
Arg1: Arginase 1
BMDM: Bone Marrow-Derived Macrophages
CAR: Chimeric Antigen Receptor
CCL2: C-C Motif Chemokine Ligand 2
CCL5: C-C Motif Chemokine Ligand 5
CCL22: C-C Motif Chemokine Ligand 22
CD: Cluster Of Differentiation
CRISPR: Clustered Regularly Interspaced Short Palindromic Repeats
CRS: Cytokine Release Syndrome
CX3CL1: C-X3-C Motif Chemokine Ligand 1
CXCL9: C-X-C Motif Chemokine Ligand 9
CXCL10: C-X-C Motif Chemokine Ligand 10
c-MYC: Myeloid Cytomatosis Oncogene Homolog
ECM: Extracellular Matrix
FcγR: Fcγ
FDA: Food And Drug Administration
GvHD: Graft-Versus-Host Disease
HER2: Human Epidermal Growth Factor Receptor 2
HIF-1α: Hypoxia-Inducible Factor-1α
HLA: Human Leukocyte Antigen
hLT-BMDM: Human Long-Lived Bone Marrow-Derived Macrophages
hPSC: Human Pluripotent Stem Cells
iBMDM: Immortalized Bone Marrow-Derived Macrophages
iCasp9: Inducible Caspase 9
ID3: Inhibitor Of DNA Binding 3
IFN-γ: Interferon-γ
IL-1β: Interleukin 1β
IL-10: Interleukin 10
IL-12: Interleukin 12
iMacs: Induced Macrophages
iNOS: Inducible Nitric Oxide Synthase
iPSCs: Induced Pluripotent Stem Cells
LPS: Lipopolysaccharides
LT-BMDM: Long-Lived Bone Marrow-Derived Macrophages
LNP: Lipid Nanoparticle
Mcl-1: Myeloid Cell Leukemia-1
MHC: Major Histocompatibility Complex
MMPs: Matrix Metalloproteinases
mRNA: Messenger RNA
NADPH: Nicotinamide Adenine Dinucleotide Phosphate
NF-κB: Nuclear Factor Kappa-B
NO: Nitric Oxide
NOX2: NADPH Oxidase 2
PBMC: Peripheral Blood Mononuclear Cells
PD-1: Programmed Death 1
PD-L1: Programmed Cell Death Ligand 1
PHD: Prolyl Hydroxylase
pHLIP: pH-Low Insertion Peptide
PMA: Phorbol-12-Myristate-13-Acetate
PRRs: Pattern Recognition Receptors
RAC1: Ras-Related C3 Botulinum Toxin Substrate 1
ROS: Reactive Oxygen Species
SIRPα: Signal Regulatory Protein α
TAAs: Tumor-Associated Antigens
TAMs: Tumor-Associated Macrophages
TCF1: T-Cell Factor 1
TGF-β: Transforming Growth Factor Beta
THP-1: Human Monocytic Leukemia Cells
TILs: Tumor-Infiltrating Lymphocytes
TIM-3: T-Cell Immunoglobulin Domain And Mucin Domain-3
TLR4: Toll-Like Receptor 4
TME: Tumor Microenvironment
TNF-α: Tumor Necrosis Factor α
Tpex: Precursors Of Exhausted T Cells
Treg: Regulatory T Cells
VD3: 1,25-Dihydroxyvitamin D3
